# Sub-Minute Analysis of Lactate from a Single Blood Drop Using Capillary Electrophoresis with Contactless Conductivity Detection in Monitoring of Athlete Performance

**DOI:** 10.3390/molecules26195817

**Published:** 2021-09-25

**Authors:** Petr Kubáň, Věra Dosedělová, Kert Martma, Indrek Rannama, Karmen Reinpold, Ruth Shimmo

**Affiliations:** 1 Department of Bioanalytical Instrumentation, Institute of Analytical Chemistry, Czech Academy of Sciences, 60200 Brno, Czech Republic; vera.dosedelova@gmail.com; 2 Central European Institute of Technology and Faculty of Science, Masaryk University, 62500 Brno, Czech Republic; 3 School of Natural Sciences and Health, Tallinn University, 10120 Tallinn, Estonia; kertmartma@gmail.com (K.M.); rannama@tlu.ee (I.R.); karmen.reinpold@tlu.ee (K.R.)

**Keywords:** capillary electrophoresis, lactate, blood plasma separation, CE-C^4^D, training assessment

## Abstract

A simple and fast method for the analysis of lactate from a single drop of blood was developed. The finger-prick whole blood sample (10 µL) was diluted (1:20) with a 7% (*w*/*v*) solution of [tris(hydroxymethyl)methylamino] propanesulfonic acid and applied to a blood plasma separation device. The device accommodates a membrane sandwich composed of an asymmetric polysulfone membrane and a supporting textile membrane that allows the collection of blood plasma into a narrow glass capillary in less than 20 s. Separated and simultaneously diluted blood plasma was directly injected into a capillary electrophoresis instrument with a contactless conductivity detector (CE-C^4^D) and analyzed in less than one minute. A separation electrolyte consisted of 10 mmol/L l-histidine, 15 mmol/L dl-glutamic acid, and 30 µmol/L cetyltrimethylammonium bromide. The whole procedure starting from the finger-prick sampling until the CE-C^4^D analysis was finished, took less than 5 min and was suitable for monitoring lactate increase in blood plasma during incremental cycling exercise. The observed lactate increase during the experiments measured by the developed CE-C^4^D method correlated well with the results from a hand-held lactate analyzer (R = 0.9882). The advantage of the developed CE method is the speed, significant savings per analysis, and the possibility to analyze other compounds from blood plasma.

## 1. Introduction

Blood lactate concentration is one of the most often measured parameters during clinical exercise testing as well as during performance testing of athletes. Lactate is one of the substances produced by cell metabolism, with the highest level of production occurring in the muscles, where it accumulates before it is released into the blood stream. Lactate levels differ slightly for venous and arterial blood. Normal lactate ranges for venous blood are 0.5 to 2.2 mmol/L and 0.5 to 1.6 mmol/L for arterial blood [1]. Serum lactate levels usually increase due to an inadequate amount of oxygen in cells and tissues (hypoxia) and clinically may indicate, among others, severe dehydration, heart failure, respiratory failure, hemorrhage, ketoacidosis, severe infections, shock, and liver disease [2,3]. Increased lactate concentration is, however, not a specific diagnostic marker, as it may indicate a wide range of conditions. For instance, blood lactate monitoring has been found useful for in-hospital mortality risk assessment for patients admitted acutely to the hospital [4].

Targeted measurement of plasma lactate concentrations can be useful in athletes during training, mainly for the determination of the lactate threshold (LT) and maximal lactate steady state (MLSS). The LT corresponds to the exercise intensity (workload) associated with a substantial increase in blood lactate during an incremental exercise test [5]. The LT is perhaps the best single predictor of endurance performance [6]. Aerobic training has been shown to improve a LT, with a concomitant improvement in endurance performance [7]. A “good” or “trained” athlete will exhibit a higher LT than a “poor” or “untrained” one at any absolute intensity above resting. In a typical incremental exercise setting, the intervals at which the power is increased range from 1 to 5 min. At the end of each period, a blood sample should be taken and lactate concentration analyzed. It is therefore necessary to have a suitable analytical procedure able to acquire and analyze the blood sample within the same timeframe (1–5 min). The routinely used methods for lactate determination are based, for instance, on enzymatic reactions and photometric absorbance measurements [8] that require rather large sample volumes and are time consuming. Other methods are available, such as amperometric sensors [9], high performance liquid chromatography (HPLC) [10], or gas chromatography (GC) [11]. Unfortunately, none of the above methods provides the required speed for quick lactate monitoring. There are hand-held lactate analyzers available from different producers that can be quick enough. A comparison of six such devices was provided by Bonaventura et al. [12] who concluded that no single portable analyzer is both highly accurate and reliable throughout the range of ~1–23 mmol/L. However, since the biological variation of blood lactate concentrations exceeds the analytical variation, the results suggest that any of the evaluated analyzers could be used over time to reliably derive blood LTs and prescribe training intensity within an individual. Different analyzers from the same manufacturer can be used interchangeably to do so, but serve only to roughly estimate the lactate values.

One of the alternative candidate analytical techniques for the analysis of lactate is capillary electrophoresis (CE). CE can provide very fast analyses, often under one minute, if the separation system is carefully optimized. Lactate is a small, ionizable molecule that can be separated and detected by CE with various detection schemes, mostly UV-Vis and conductometric detection. While there are various accounts of using CE with indirect UV-Vis detection of lactate for blood or blood plasma sample analysis [13,14,15], the use of contactless conductivity detection (C^4^D) for lactate analysis in blood plasma is rare, but may offer some advantages over the UV-Vis detection [16] because lactate does not absorb in the UV-Vis range. While analysis of blood plasma or serum by CE is possible (usually after sufficient dilution), the analysis of whole blood is not feasible due to the presence of red blood cells and other cellular materials that clog the separation capillary or cause severe variation of electroosmotic flow and consequently variation of migration times. For fast lactate screening in blood, finger-prick sampling is preferable, because it can be done easily and quickly at short intervals between various power loads during incremental exercise. Unfortunately, analysis and separation of blood plasma from such a small sample is difficult using conventional laboratory equipment. In the work by Pormsila et al. [16], which is the sole article that has described the determination of lactate in blood plasma during exercise by CE-C^4^D, heparinized blood plasma samples were obtained from venous blood by centrifugation and protein precipitation prior to CE analysis. All samples were thus analyzed after the incremental exercise setup has been performed. Depending on the storage conditions and the analysis timeframe, lactate levels can increase in stored blood samples as shown by Oyet et al. [17]; thus, emphasizing the apparent need for direct and fast analysis methods.

In this work, we attempted to develop a simple and fast procedure for at-site sampling of capillary blood samples from a finger-prick blood drop, subsequent blood plasma separation, and analysis, allowing a maximum of 5 min between the measurements. In our previous research, we have constructed a simple blood plasma separation device that is able to separate blood plasma from very small volumes of whole blood (10–20 µL) in a very short time, typically 20 s [18]. This device was previously tested for analysis of formate in blood of patients intoxicated with methanol with promising results. In this work, we demonstrate another suitable application of the developed device, the analysis of lactate in monitoring athlete performance. We show that sampling and CE analysis of lactate is possible in less than 5 min (total time from finger-prick puncture, sample aspiration, blood plasma separation, and CE analysis). The observed lactate increase during the incremental rise of power using our method correlated well with a hand-held lactate analyzer (R = 0.9882). The advantage of the developed CE method is the significant savings per analysis and the possibility to analyze other compounds from blood plasma.

## 2. Results and Discussion

### 2.1. Selection of Separation Conditions

The initial selection of CE parameters and background electrolyte (BGE) was taken from our previous publication [19], in which formate and lactate could be efficiently separated in diluted blood plasma. The separation electrolyte consisted of 10 mmol/L l-histidine (HIS), 15 mmol/L dl-glutamic acid (GLU), and 30 µmol/L cetyltrimethylammonium bromide (CTAB), with final pH of 4.6. The lactate peak could be analyzed in about 3 min using a separation capillary of a total length of 50 cm and an effective length of 35 cm. Since the resolution of lactate from the closest peak present in blood plasma samples (formate) was large (R = 13.7), the separation capillary was shortened to 25 cm total, 15 cm effective length, yielding a subminute analysis of lactate from blood plasma. Additionally, the high voltage was increased to −18 kV compared to previous publications. The high voltage can be increased; however, care should be taken to monitor possible Joule heating from excessive currents at high voltages. In our case the separation current was 6.1 µA, which was acceptable. Another means to increase the separation of anions in co-electroosmotic mode is to increase the concentration of CTAB. However, since the analysis speed was sufficient no modification to the original BGE composition was made. 

**Figure 1:**
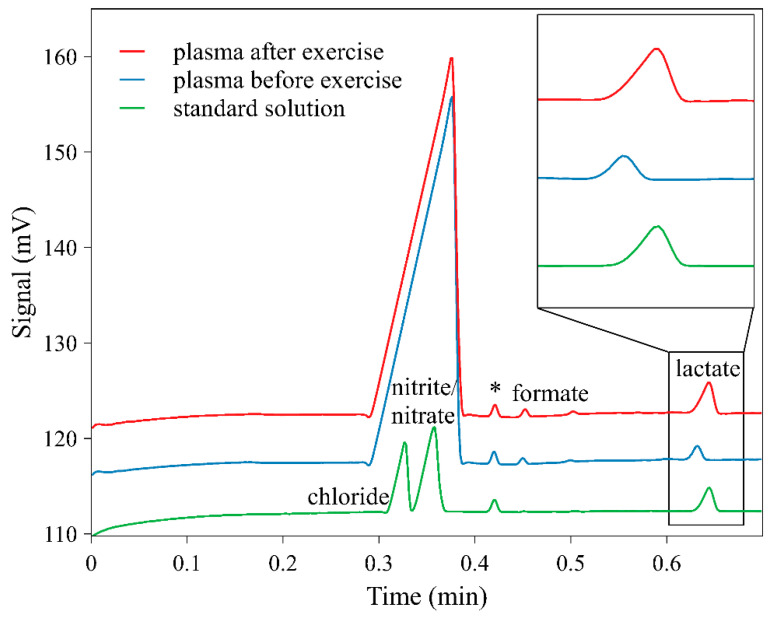
Separation of a standard solution of chloride, nitrate, nitrite, and lactate and blood plasma samples separated using the blood plasma separation device. Separation conditions: BGE 10 mM HIS, 15 mM GLU, 30 µM CTAB, pH 4.6, injection: hydrodynamic, 30 s/10 cm, separation voltage −18 kV, separation current 6.1 µA. (*) peak is the impurity from TAPS diluent.

With these conditions, the migration time of lactate was about 0.7 min, while the resolution of the closest peak of formate decreased to R = 7.5. Subminute separation was very useful for fast screening of lactate during the exercises. Figure 1 shows an example of the electropherograms of a standard solution of inorganic anions and lactate, and two blood plasma samples prepared with the developed device and method—one before and the other one after the exercise, demonstrating the ability to monitor an increase in lactate concentration.

### 2.2. Comparison of the Blood Plasma Separation Device with Centrifugation 

To verify that the performance of the plasma separation device is comparable to centrifugation, which is commonly used in routine practice, the diluted whole blood was split into four aliquots of 200 µL (10 µL sample + 190 µL 7% TAPS). First two aliquots were applied to the blood plasma separation device. The other two aliquots were centrifuged at 2350 *g* for 10 min. The obtained plasma samples were collected, respectively and analyzed by CE-C^4^D. The average concentrations of lactate in plasma obtained by the two methods were 3.5 mM and 3.8 mM, respectively. The values were not statistically different. These experiments confirmed that the developed device provides a comparable performance to centrifugation and that there are no losses of lactate during the separation process.

### 2.3. Analytical Parameters of the Method 

#### 2.3.1. Intra-Day and Inter-Day Precision of Migration Times and Peak Areas

The analytical parameters of the method are listed in Table 1. The repeatability of the results (intra-day precision) was evaluated by repeatedly analysing a standard solution of lactate at the concentration level of 100 µmol/L. The repeatability of migration times (n = 10) was 0.7% RSD, while the repeatability of peak areas was less than 2.7%. The same procedure was also performed with a freshly separated blood plasma sample. The repeatability of migration times (n = 10) was 0.9% RSD, while the repeatability of peak areas was 2.9%. These results show that the repeatability is comparable between the standards and blood plasma samples. The inter-day repeatability was obtained as a result of 3-day measurements using the same standard and separated blood plasma sample solutions. The samples were stored in a deep freezer at −20 °C overnight. The inter-day precision, expressed in RSD was only slightly worse than the intra-day precision, for the migration time it was 1.3% RSD, and for the peak areas it was 3.5%. The results demonstrate that the method is sufficiently robust and the samples and standards can be analyzed repeatedly even on subsequent days. 

#### 2.3.2. Recoveries

The recoveries were evaluated by spiking the blood plasma samples with lactate at two levels: 100 and 600 μmol/L, corresponding to the low and high concentrations expected in the samples. The concentrations after adding the lactate standard were evaluated from the calibration curve and the found concentration was compared with the known added concentration. The recoveries were 105% and 103% for the 100 μmol/L and 600 μmol/L levels, respectively (Table 1).

#### 2.3.3. Calibration, Limit of Detection, Limit of Quantitation

The developed method is characterized by a linear dependence of the analytical signal (peak area) on the concentration in the range of 0–800 μmol/L. The R-square for lactate was ≥ 0.999. The limit of detection (LOD) and limit of quantification (LOQ) were calculated from the standard deviation of the calibration response (σ) and the slope (s) of the calibration dependence (LOD = 3.3 (σ/s), LOQ = 10 (σ/s)). The LOD and LOQ were 6 and 19 μmol/L, respectively (Table 1).

### 2.4. Correlation between the Developed CE Method and a Hand-Held Device

**Figure 2:**
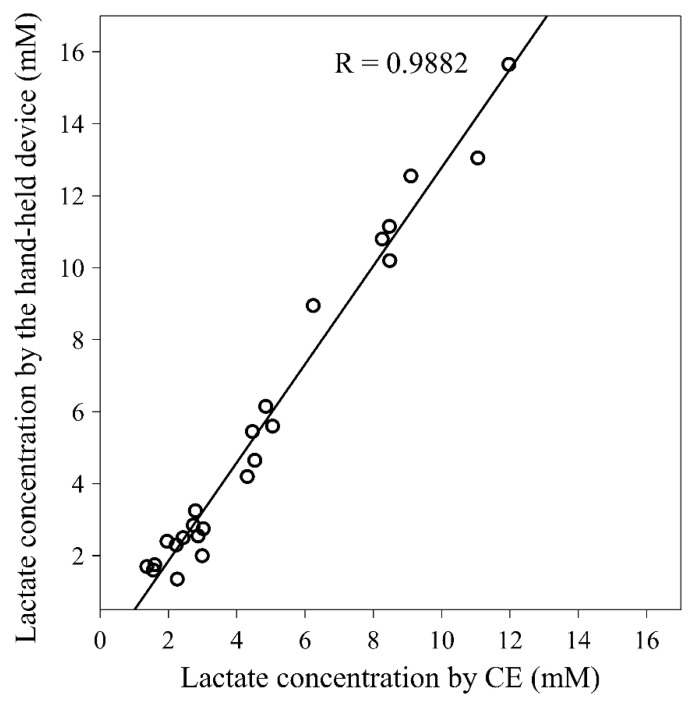
Correlation of the CE method with the hand-held lactate analyzer.

All blood samples that were collected by the finger-prick sampling method were also simultaneously analyzed by the hand-held device Lactate Scout + (SensLab GmbH). The data obtained from the developed method were compared with the data from the hand-held device. Figure 2 shows the correlation of the results. The Pearson correlation coefficient, R, was calculated to be 0.9882, which means that there was an excellent correlation between our developed method and the analysis by a hand-held device. One can also notice that the results obtained by CE are slightly lower than those obtained by the hand-held device. We do not know the exact reason for these discrepancies at the moment, it can be that while the CE method uses blood plasma, whole blood is injected into the hand-held device. Further detailed study would be required, including a third, certified laboratory method to find the reason for these differences, however, this was not within the scope of the work here. 

### 2.5. Content of Lactate in Blood Plasma during Exercise

**Figure 3:**
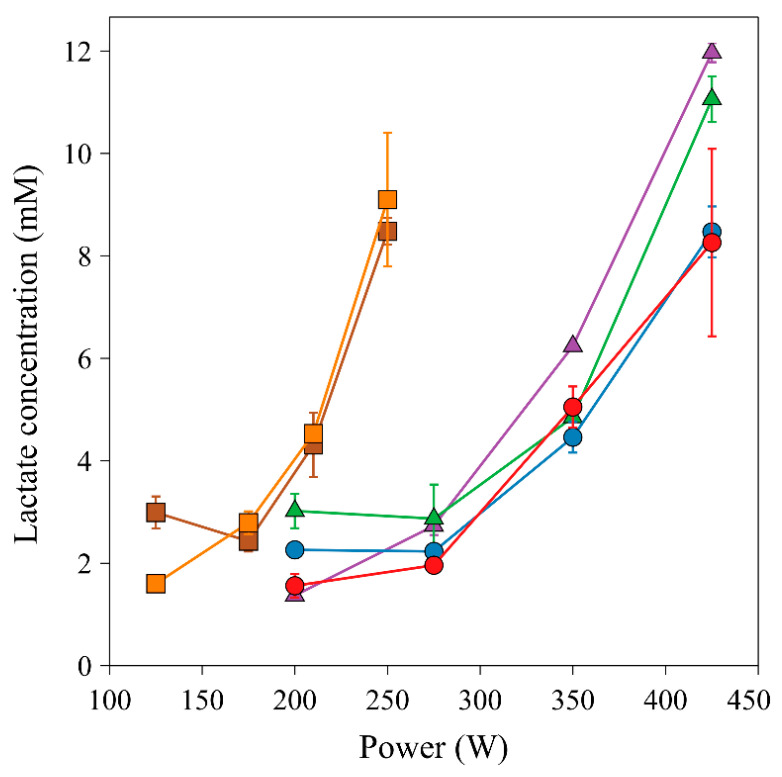
Graph of lactate concentration in blood plasma at 4 different power settings. Separation conditions: BGE 10 mM HIS, 15 mM GLU, pH 4.6, injection: hydrodynamic, 30 s/10 cm, separation voltage −18 kV, separation current 6.1 µA. (☐-untrained cyclist, △,◯-trained cyclists, different color is for values measured on different days).

Figure 3 shows the results of the blood plasma lactate analysis by CE-C^4^D obtained from three volunteers. The volunteers were asked to perform cycling using a professional ergometer Cyclus 2 (Avantronic, Cyclus 2, Leipzig, Germany), equipped with cyclists personal racing bicycles, at four different power settings on two subsequent days. The power levels lasted 5 min and were chosen to describe 4 different intensities used in regular aerobic training: lower than LT, near LT, near MLSS, and maximal aerobic power at a maximal oxygen consuption (VO_2max_) level. A one-minute recovery pause was used between power levels for lactate sampling. Two well-trained (age 37 and 45 years, body mass 79.4 and 78.8 kg, height 1.85 and 1.84 m) and one untrained (age 48 years, body mass 78.0, height 1.77 m) cyclists [20] were incorporated in the experimental part. The power for the two well-trained cyclists was set at 200 W, 275 W, 350 W, and 425 W, while the power for the untrained cyclist was set to 125 W, 175 W, 200 W, and 250 W. It is obvious from the graph in Figure 3 that the performance of each of the volunteers is rather consistent between days and that although the absolute levels of lactate differ among persons, there is a similar increase of lactate at the selected power setting (last two values) that were considered near MLSS and VO_2max_ levels. Apparently, the first two power settings generated no significant elevation of lactate in blood for all volunteers. Thus, it is demonstrated that the absolute levels of lactate are not as important as the trend and lactate increase and that they depend on the “fitness” level of each person. The developed method demonstrated that it is simple and fast enough to analyze lactate during the incremental exercise setup and is able to provide reliable results.

## 3. Materials and Methods

### 3.1. Chemicals and Reagents

All chemicals were of reagent grade and DI water (Purite, Neptune, Watrex, Prague, Czech Republic) was used for stock solution preparation and dilutions. 10 mM stock solutions of inorganic anions were prepared from their sodium salts (chloride, nitrate, nitrite, all from Pliva-Lachema, Brno, Czech Republic). Lithium lactate was from Pliva Lachema, Brno, Czech Republic. BGE for CE measurements was prepared daily by diluting 200 mM stock solution of L-histidine (HIS, Sigma-Aldrich, Steinheim, Germany) and 50 mM solution of dl-glutamic acid (GLU, Sigma, Steinheim, Germany) to the required concentration. Cetyltrimethylammonium bromide (CTAB, Sigma-Aldrich, Steinheim, Germany) was prepared as a 10 mM stock solution in 5% acetonitrile and was added to the BGE to yield the final concentration of 30 µM. A 7% (*w*/*v*) solution of [tris(hydroxymethyl)methylamino] propanesulfonic acid (TAPS, Sigma-Aldrich, Steinheim, Germany) was prepared by dissolving 0.7 g of TAPS in 10 mL of DI water.

### 3.2. CE Instrumentation

A purpose-made CE instrument was built in house. The instrument consisted of a negative high-voltage power supply (Spellman UM20N4, Spellman, Pulborough, UK) with two Pt/Ir wire electrodes (of 0.5 mm outer diameter (o.d.), 5 cm long). The electrodes were inserted into two 20 mL glass vials (1530-0201, Fisher Scientific, Pardubice, Czech Republic) with a hole made in the lid containing the BGE. A fused-silica capillary (Microquartz GmbH, Munich, Germany, inner diameter (i.d.) 50 μm, o.d. 365 μm) had a 25 cm total length and 15 cm effective length (to the detector). A new capillary was rinsed sequentially with 0.1 mol/L NaOH (30 min), DI water (30 min), and BGE (30 min) by vacuum using a 5 mL syringe. The sample injection was performed manually by elevating the injection capillary end to the height of 10 cm for 30 s. After each analysis, the capillary was flushed with BGE for 1 min to maintain the reproducibility of migration times. After the last analysis during the working day, the capillary was rinsed with DI water (10 min) and air (5 min). The separations were carried out at ambient temperature with the voltage of −18 kV, applied at the injection side. 

For detection, a custom-made C^4^D detector (Version 5.06, ADMET s.r.o., Prague, Czech Republic) operating at a frequency of 2 MHz and voltage 50 V_p-p_ was used.

### 3.3. Blood Plasma Separation Device

**Figure 4:**
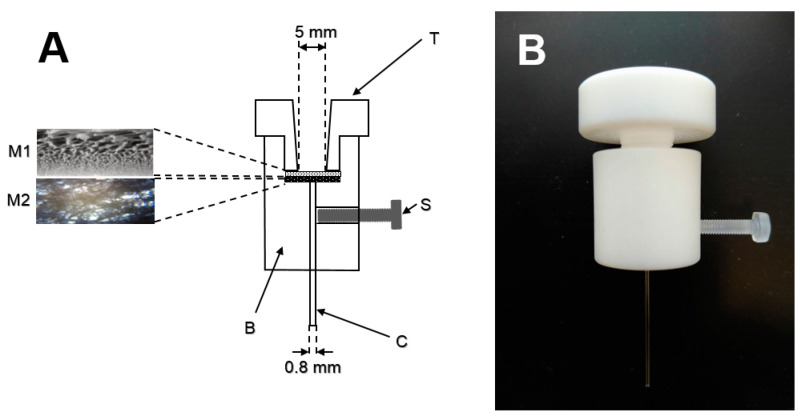
A schematic of the blood plasma separation device (**A**) with the photographs of the membrane structures. A photograph of the assembled device (**B**).

The blood plasma separation device was constructed by CNC machining. Its schematic diagram and photograph are shown in Figure 4. The device consists of two parts made from polytetrafluorethylene (PTFE). The top part (T) has a shape of a large screw (diameter 10 mm, length 13 mm) with a 5 mm conical hole in the center for blood sample introduction. The cylindrical bottom part (B) has a 7 mm deep, 10 mm diameter hole to accommodate the separation and supporting membranes (M1, M2). In the bottom, a 1 mm hole is drilled for insertion of the blood plasma collection capillary (C, 41 mm length, 0.56 mm i.d., 0.8 mm o.d., Microcapillary tube Drummond Microcaps^®^, Drummond Scientific Company, Broomall, PA, USA). The collection capillary is affixed to its position, gently touching the supporting membrane (M2) using a screw (S). The separation and supporting membranes are tightly sealed in place by the top PTFE part.

A Vivid^TM^ GR plasma separation membrane (Pall Corporation, East Hills, NY, USA) was used. The membrane thickness is approximately 330 µm and the capacity for blood filtration is 40–50 µL per cm^2^ membrane area. Figure 4 (left) shows a schematic of the membrane (Pall Corporation, East Hills, NY, USA). The membrane was cut into a circle using an 8 mm diameter hollow punch tool. Under the separation membrane, a supporting textile membrane (Non-woven Wipe, CLEANTEX, Prostejov, Czech Republic) with the same diameter was placed. This membrane (schematic of the membrane structure is shown in Figure 4) enhanced the blood plasma flow and allowed fast collection of plasma into a 10 μL collection capillary (C). The collection capillary provided an additional hydrostatic capillary force to remove blood plasma from the set of membranes.

### 3.4. Hand-Held Lactate Analyzer

A commercial hand-held analyzer (Lactate Scout+, SensLab GmbH, EKF-diagnostic, Barleben, Germany) was used as a reference to compare the performance of the developed CE-C^4^D method. The device has the measurement range of 0.5–25.0 mmol/L, coefficient of variation of 3–8% and is able to analyze 0.5 µL of whole blood. The results are typically obtained in less than 1 min including sampling and analysis. 

## 4. Conclusions

In this work, we have developed a fast, simple, and reproducible method for analyzing lactate in single blood drop samples as small as 10 µL. The procedure includes all necessary steps, such as dilution and blood plasma separation, yielding a ready to inject blood plasma sample. The analysis of the samples by CE-C^4^D is very fast, the separation takes about 45 s, so that the whole procedure, from the finger-prick sampling until the analysis result is obtained, takes less than 5 min. The method was tested by measuring lactate during incremental cycling exercise when testing the cyclist performance. The developed method has a potential to be used also in clinical settings, for instance, to analyze the concentration of lactate in critically ill patients. 

## Figures and Tables

**Table 1:** Analytical parameters of the developed method.

Parameter	Lactate
Linearity (μmol/L)	0–800
Slope	0.013
Intercept	0.1361
R-squared	0.9993
LOD (μmol/L)	6
LOQ (μmol/L)	19
**Intra-day precision ^1^, CV (%):**	
100 μmol/L standard	0.7 (2.7) ^2^
Blood plasma	0.9 (2.9) ^2^
**Inter-day precision ^1^, CV (%):**	
100 μmol/L standard	1.3 (3.5) ^2^
Blood plasma	1.3 (3.5) ^2^
**Recovery (%):**	
100 μmol/L standard added	105%
600 μmol/L standard added	103%

^1^ n = 10 for intra-day precision; n = 30 for inter-day precision, ^2^ the value is for migration time, the value in parenthesis is for peak area.

## Data Availability

Not applicable.

## Sample Availability

Samples of the compounds are not available from the authors.

## References

[1] Richard A., Kress T. (2009). Measuring serum lactate. Nurs. Crit. Care.

[2] Fischbach F.T., Dunning M.B. (2008). A Manual of Laboratory and Diagnostic Tests.

[3] Kee J.L. (2002). Laboratory and Diagnostic Tests with Nursing Implications.

[4] Kruse O., Grunnet N., Barfod C. (2011). Blood lactate as a predictor for in-hospital mortality in patients admitted acutely to hospital: A systematic review. Scand. J. Trauma Resusc. Emerg. Med..

[5] Svedahl K., MacIntosh B.R. (2003). Anaerobic Threshold: The Concept and Methods of Measurement. Can. J. Appl. Physiol..

[6] Goodwin M.L., Harris J.E., Hernández A., Gladden L.B. (2007). Blood Lactate Measurements and Analysis during Exercise:A Guide for Clinicians. J. Diabetes Sci. Technol..

[7] Kumagai S., Tanaka K., Matsuura Y., Matsuzaka A., Hirakoba K., Asano K. (1982). Relationships of the anaerobic threshold with the 5 km, 10 km, and 10 mile races. Eur. J. Appl. Physiol. Occup. Physiol..

[8] Alderman J.A., Cross R.E. (1977). Adaptation to the centrifugal analyzer of an enzymatic method for the measurement of lactate in plasma and cerebrospinal fluid. Clin. Chem..

[9] de Keijzer M.H., Brandts R.W., Brans P.G.W. (1999). Evaluation of a biosensor for the measurement of lactate in whole blood. Clin. Biochem..

[10] Hasegawa H., Fukushima T., Lee J.A., Tsukamoto K., Moriya K., Ono Y., Imai K. (2003). Determination of serum d-lactic and l-lactic acids in normal subjects and diabetic patients by column-switching HPLC with pre-column fluorescence derivatization. Anal. Bioanal. Chem..

[11] Paik M.J., Cho E.Y., Kim H., Choi S., Ahn Y.H., Lee G. (2008). Simultaneous clinical monitoring of lactic acid, pyruvic acid and ketone bodies in plasma as methoxime/tert-butyldimethylsilyl derivatives by gas chromatography-mass spectrometry in selected ion monitoring mode. Biomed. Chromatogr..

[12] Bonaventura J.M., Sharpe K., Knight E., Fuller K.L., Tanner R.K., Gore C.J. (2015). Reliability and accuracy of six hand-held blood lactate analysers. J. Sci. Med. Sports.

[13] Dolnik V., Dolnikova J. (1995). Capillary zone electrophoresis of organic acids in serum of critically ill children. J. Chromatogr. A.

[14] Oefner P.J. (1995). Surface-charge reversed capillary zone electrophoresis of inorganic and organic anions. Electrophoresis.

[15] Markuszewski M.J., Szczykowska M., Siluk D., Kaliszan R. (2005). Human red blood cells targeted metabolome analysis of glycolysis cycle metabolites by capillary electrophoresis using an indirect photometric detection method. J. Pharm. Biomed. Anal..

[16] Pormsila W., Morand R., Krahenbuhl S., Hauser P.C. (2011). Quantification of plasma lactate concentrations using capillary electrophoresis with contactless conductivity detection. Electrophoresis.

[17] Oyet C., Okongo B., Onyuthi R.A., Muwanguzi E. (2018). Biochemical changes in stored donor units: Implications on the efficacy of blood transfusion. J. Blood. Med..

[18] Ďurč P., Foret F., Kubáň P. (2018). Fast blood plasma separation device for point-of-care applications. Talanta.

[19] Kubáň P., Foret F., Bocek R. (2013). Capillary electrophoresis with contactless conductometric detection for rapid screening of formate in blood serum after methanol intoxication. J. Chromatogr. A.

[20] De Pauw K., Roelands B., Cheung S.S., De Geus B., Rietjens G., Meeusen R. (2013). Guidelines to classify subject groups in sport-science research. Int J. Sports Physiol Perform..

